# Effectiveness of exercise training in people with non-cystic fibrosis bronchiectasis with and without COPD

**DOI:** 10.3389/fresc.2026.1764160

**Published:** 2026-03-06

**Authors:** Michele Vitacca, Mara Paneroni, Antonio Spanevello, Mauro Carone, Giuseppina Cassetti, Cinzia Lastoria, Mauro Maniscalco, Armando Capelli, Nicolino Ambrosino

**Affiliations:** 1 Istituti Clinici Scientifici Maugeri IRCCS, Respiratory Rehabilitation of the Institute of Lumezzane, Brescia, Italy; 2 Istituti Clinici Scientifici Maugeri IRCCS, Respiratory Rehabilitation of the Institute of Tradate, Varese, Italy; 3 MACRO, University of Insubria, Varese, Italy; 4 Istituti Clinici Scientifici Maugeri IRCCS, Respiratory Rehabilitation of the Institute of Bari, Bari, Italy; 5 Department of Medical and Surgical Science, University of Foggia, Foggia, Italy; 6 Istituti Clinici Scientifici Maugeri IRCCS, Respiratory Rehabilitation of the Institute of Milano, Milano, Italy; 7 Istituti Clinici Scientifici Maugeri IRCCS, Respiratory Rehabilitation of the Institute of Pavia, Pavia, Italy; 8 Istituti Clinici Scientifici Maugeri IRCCS, Respiratory Rehabilitation of the Institute of Telese, Benevento, Italy; 9 Department of Clinical Medicine and Surgery, University of Napoli Federico II, Napoli, Italy; 10 Istituti Clinici Scientifici Maugeri IRCCS, Respiratory Rehabilitation of the Institute of Veruno, Novara, Italy; 11 Istituti Clinici Scientifici Maugeri IRCCS, Respiratory Rehabilitation of the Institute of Montescano, Pavia, Italy

**Keywords:** COPD, dyspnea, exercise tolerance, health status, pulmonary rehabilitation

## Abstract

**Background and aim:**

Non-Cystic Fibrosis (CF) bronchiectasis is associated with reduced exercise tolerance and symptoms such as dyspnea and fatigue, impairing functional capacity and limiting physical activity. In addition to airway clearance techniques, pulmonary rehabilitation (PR), including aerobic and resistance training, is recommended in these individuals. This retrospective, multicentric study covering a ten-year period compared in non-CF bronchiectasis people with and without COPD the effectiveness of PR, including exercise training, on exercise tolerance (primary objective) and other patient-centered outcomes.

**Measurements:**

Before and after PR, the following assessments had been performed: Six-minute walking test (6MWT), Barthel Index (BI), Barthel Index dyspnea (BId), COPD assessment test (CAT), Short physical performance battery (SPPB).

**Results:**

125 participants without and 1,346 with concomitant COPD were studied. Non-COPD bronchiectasis was more prevalent in females, showed a higher association with asthma, whereas Chronic Respiratory Failure (CRF) and pneumonia were more prevalent in COPD-related bronchiectasis, resulting in more prevalent referral from acute care hospitals or need for oxygen therapy. All baseline outcome measures, except CAT, were worse in people with concomitant COPD. After PR, 6MWT improved significantly in both groups [by 39 (11–70) and 40 (10–75) meters for non-COPD and COPD-related bronchiectasis, respectively, *p* = 0.7469], without any between-group difference. The other outcome measures also improved significantly in both groups. The proportions of participants reaching the minimal clinically important difference in assessed outcomes were not significantly different between the studied populations.

**Conclusion:**

Pulmonary rehabilitation, including exercise training, yields l equivalent benefits in exercise capacity and patient-reported outcomes in non-CF bronchiectasis people with or without COPD. Our results suggest the routine clinical provision of PR to people with non-CF bronchiectasis, regardless of concomitant COPD.

## Introduction

Non-cystic fibrosis (CF) bronchiectasis is a chronic lung condition characterized by bronchial dilatation and inflammation with daily cough, sputum, and recurrent exacerbations. The reported prevalence in the general population ranges from 52.3 to over 1,000 cases per 100,000 people (1). In Italy, the prevalence and incidence of non-CF bronchiectasis in primary care in 2015 were 163.0 and 16.3 per 100,000 person-years, respectively. After excluding people with a diagnosis of either asthma or COPD, the prevalence and incidence were found to be 130 and 11.1 cases per 100,000 person-years, respectively (2). Conditions associated with non-CF bronchiectasis include gastroesophageal reflux disease (47%), asthma (29%), and chronic obstructive pulmonary disease (COPD) (20%), up to 38% of cases being idiopathic (3).

Non-CF bronchiectasis is associated with reduced exercise tolerance and symptoms such as cough, dyspnea, and fatigue, which impair functional capacity and limit physical activity (4). Pharmacological and non-pharmacological treatments as airway clearance techniques and pulmonary rehabilitation including endurance and resistance training, are recommended (5, 6). Given the association between non-CF bronchiectasis and COPD and the recognized effectiveness of exercise training in individuals with COPD (7), we wondered whether a standard in-patient rehabilitation program including endurance training would have given similar results in the two populations. The Italian healthcare system offers various rehabilitation settings for people with COPD and other chronic respiratory diseases. The inpatient setting is considered appropriate in cases of clinical complexity (i.e., following a recent exacerbation) (8).

Therefore, the aim of this retrospective, multicentric study covering a ten-year period was to compare the effectiveness of an in-patient pulmonary rehabilitation program, including exercise training, in non-CF bronchiectasis individuals with or without COPD. The primary outcome was exercise tolerance as assessed by the six-minute walk distance test (6MWT). Secondary outcomes were dyspnea and health status.

## Methods

### Participants

The retrospective multicentric study was conducted on data on individuals with bronchiectasis diagnosis (ICD-9-CM cod = 4,940–4,941) with (COPD non-CF Bronchiectasis) or without (Non-COPD non-CF Bronchiectasis) COPD (ICD-9-CM Cod = 49,120–49,121). Others comorbidities according to ICD-9-CM were also recorded All participants had been recovered from an exacerbation of their disease, either cared for in acute care hospitals or at home by their general practitioners, and admitted for pulmonary rehabilitation between January, 1st, 2015 to December, 31st, 2024 to Rehabilitation Hospitals of the ICS Maugeri, IRCCS network (Lumezzane, Pavia, Veruno, Montescano, Tradate, Ginosa, Telese, Bari, Veruno, Milano). These hospitals share common indications, evaluation, diagnostic, and management tools as well as protocols for pulmonary rehabilitation. Ethical approval was obtained for this study (EC Lombardia 6 Prot. 18390/25; 11 March 2025). Given the retrospective nature of the study on anonymized data, the participants' permission was waived.

Exclusion criteria from the rehabilitation program had included: severe comorbidities such as oncological, neurological disorders, heart failure, recent (less than 4 months) acute ischemic cardiovascular diseases with an unstable status, inability or refusal to perform the program, and participants with missing pre- or post-program data of 6MWT.

### Measurements

At admission, the following data had been recorded: demographics, anthropometrics, Comorbidity Index of the Cumulative Illness Rating Scale (CIRS) (9), Body-Mass Index (BMI), provenience (hospital or home), length of hospital stay (LoS), concomitant chronic respiratory failure (CRF).

Before and after the program, the following assessments were performed:

Exercise tolerance by the 6MWT according to standard (10). Data are shown as meters and percentages of predicted values (11). In clinical practice, a change in 6MWT ≥ 24.5 meters should be interpreted as the minimal clinically important difference (MCID) in adults with bronchiectasis (12).
Functional disability by the Barthel Index (BI) (13).
Dyspnea by the Barthel Index dyspnea (BId) (14). The MCID has been defined as a 9-point reduction for COPD individuals without and as a 12-point reduction for individuals with CRF, respectively (15).
Health status by the COPD assessment test (CAT) (16). A two-point reduction in score has been reported as the MCID (17).
Short physical performance battery (SPPB) (18). An improvement of at least 1 point for SPPB is reported as the MCID (19).

### Pulmonary rehabilitation

**Figure 1:**
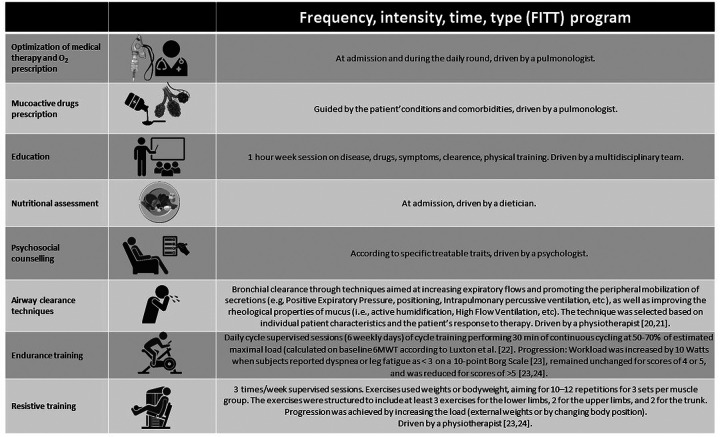
The rehabilitation program. Icons from Biorender (https://www.biorender.com/).

A full explanation of the rehabilitation program is described in Figure 1. All hospitals are part of the same network, heads of wards and physiotherapy services hold regular meetings to align protocols to be followed, according to guidelines. Our in-hospital program is supervised by multidisciplinary teams of trained and experienced chest physicians, nurses, physiotherapists, dieticians, and psychologists, dedicated full-time to pulmonary rehabilitation as previously described (25). In brief, the in-hospital program starts within 2 days since admission, after baseline evaluations, and includes daily supervised sessions of physical training, according to international guidelines (26, 27).

In addition to airway clearance techniques when required (20, 21), the program also includes optimization of medications, education, nutritional programs, and psychosocial counseling when appropriate. The duration of daily activities is 2–3 h. The program is conducted in Gym Rooms with full availability of safety tools (e.g., CPR).

### Statistical analysis

We analyzed data using the software STATA 12.4 (Stata Corp LLC, College Station, TX, USA). We tested data normality using the Shapiro–Wilk test. Normally distributed variables were described by mean ± standard deviation (SD), while non-normally distributed variables were described by median **(**1st and 3rd quartiles). Categorical variables were described by numbers and relative frequencies (%). We evaluated pre-to-post differences within groups using the paired t-test for normally distributed variables and the Wilcoxon test for non-parametric variables. Between group differences (COPD non CF Bronchiectasis vs. Non-COPD non CF Bronchiectasis) were evaluated using an unpaired t-test for normally distributed variables and a Mann–Whitney U-test for non-parametric ones. The significance of differences in proportions of individuals reaching the MCID was assessed by the *χ*2 function. Spearman's correlation (rho) was used to assess the relationship between variables. Values of rho < 0.30 were interpreted as indicating weak, 0.30 ≤ rho < 0.50 moderate, and rho ≥ 0.50 strong associations between variables. A *p*-value <0.05 was considered significant.

## Results

In the period under observation, 2,673 out of 38.963 individuals (6.86%) with respiratory diseases admitted for pulmonary rehabilitation (53.4% males; age = 71.59 ± 10.93 years; BMI = 25.82 ± 6.57 kg/m^2^; CIRS = 3.76 ± 1.98) suffered from non-CF bronchiectasis. The prevalence of non-COPD (212 individuals) and COPD concomitant bronchiectasis (2,461) was 0.54 and 6.31%, respectively (for clinical details see Supplementary Table S1).

Out of these 2,673, we analyzed data from 1,471 participants with available data of pre and post-program 6MWT (125/212 without and 1,346/2,461 with COPD). The contribution to the sample size by the different centers was: Ginosa 6.85%; Milano 6.10%; Bari 20.43%; Pavia 4.08%; Lumezzane 13.02%; Montescano 6.10%; Telese 4.0%; Tradate 34.56%; Veruno 4.86%. The baseline characteristics of the populations in the study are shown in Table 1.

**Table 1:** Anthropometric, demographic, physiological, and clinical characteristics of populations in study.

Variable	Non-COPD Bronchiectasis *n* = 125	COPD Bronchiectasis *n* = 1,346	*P*-value
Male, *n* (%)	42 (33.6)	697 (51.78)	**<0**.**001**
Age, years	71 (65–78)	72 (68–75)	0.7380
BMI, kg/m^2^	26.31 ± 5.79	25.78 ± 6.63	0.6378
LoS, days	23 (21–27)	24 (21–29)	0.3959
From acute care hospitals, *n* (%)	48 (38.40)	686 (50.96)	**0**.**007**
CIRS, score	3.74 ± 2.10	3.48 ± 1.84	0.689
Asthma, *n* (%)	23 (18.40)	64 (4.75)	**<0**.**001**
OSAS *n* (%)	20 (16.0)	368 (27.34)	**0**.**006**
Hypertension, *n* (%)	90 (72.00)	1,076 (79.94)	**0**.**035**
Other CVD, *n* (%)	48 (38.40)	565 (41.97)	0.434
Diabetes *n* (%)	26 (20.80)	403 (29.94)	**0**.**031**
CRF, *n* (%)	0	428 (31.79)	**<0**.**001**
Pneumonia, *n* (%)	0	8 (0.59)	**<0**.**001**
Night/Exercise hypoxemia, *n* (%)	17 (13.60)	121 (8.9)	0.091
O_2_ supplement, *n* (%)	17 (13.60)	565 (41.97)	**<0**.**001**
ABG/night pulsossimetry, *n* (%)	97 (77.60)	1,144 (84.99)	**<0**.**001**
Lung CT scan, *n* (%)	28 (22.40)	376 (27.93)	0.183
6MWT, m	400 (301–464)	300 (210–402)	**<0**.**001**
6MWT, % prd	61.0 (43.9–71.4)	47.6 (31.1–71.4)	**<0**.**001**
BI, score	100 (79;100) (*n* = 51)	97 (85;100) (*n* = 610)	0.3446
BId, score	16 (7;28) (*n* = 55)	31 (17;49) (*n* = 760)	**<0**.**001**
CAT, score	20 (15;26) (*n* = 44)	23 (16;27) (*n* = 714)	0.3402
SPPB, score	10 (7;11) (*n* = 51)	8 (6;10) (*n* = 661)	**0**.**0135**

*n*, number of available data; ABG, Arterial Blood Gases; BMI, Body-Mass Index; LoS, Length of Stay; CIRS, Cumulative Illness Rating Scale; CRF, Chronic Respiratory Failure; CPAP, Continuous Positive Airway Pressure; CVD, CardioVascular diseases; NIV, Non-Invasive Ventilation; O_2_, Oxygen; OSAS, Obstructive Sleep Apnoea Syndrome; 6MWT, 6-Minute Walking Test; BI, Barthel Index; BId, Barthel Index Dyspnea; CAT, COPD Assessment Test; SPPB, Short Physical Performance Battery Data shown as mean (SD) or median (1st - 3rd quartiles).

The values that are statistically significant are shown in bold.

As expected, non-COPD bronchiectasis was more prevalent in females, showed a higher association with asthma, whereas CRF or pneumonia were significantly more prevalent in COPD-related bronchiectasis, resulting in more prevalent referral from acute care hospitals or need for oxygen supplement. Exercise capacity as assessed by 6MWT, dyspnea by BId and SPPB were significantly more severe in people with concomitant COPD as well. However, health status measured by CAT were not (Table 1).

Table 2 shows the baseline values and post-rehabilitation changes in assessed outcome measures. Following the program, outcome measures improved significantly at an equivalent extent in either group; only BiD improved significantly more in people with COPD-related bronchiectasis.

**Table 2:** Baseline and post program values of assessed outcomes.

Variable	Non-COPD Bronchiectasis *n* = 125		*P* value Pre to post	COPD Bronchiectasis *n* = 1,346		*P*-value pre to post	*P* value changes between groups
	Baseline	Post		Baseline	Post		
6MWT, m	400 (301;464) (*n* = 125)	430 (350;519)	**<0**.**001**	300 (210;402) (*n* = 1.345)	350 (265;440)	**<0**.**001**	0.7469
6MWT, % pred	61.0 (43.9–71.4)	70.4 (56.7–82.3)	**<0**.**001**	47.6 (31.1–71.4)	57.8 (43.9;71.3)	**<0**.**001**	0.5113
BI, score	100 (79;100) (*n* = 51)	100 (94;100)	**<0**.**001**	97 (85;100) (*n* = 610)	100 (94;100)	**<0**.**001**	0.9322
BId, score	16 (7;28) (*n* = 55)	7 (2;15)	**<0**.**001**	31 (17;49) (*n* = 760)	16 (8;29)	**<0**.**001**	**0**.**0021**
CAT, score	20 (15;26) (*n* = 44)	9.5 (8–14)	**<0**.**001**	23 (16;27) (*n* = 714)	14 (7;18)	**<0**.**001**	0.1371
SPPB, score	10 (7;11) (*n* = 51)	11 (9–12)	**<0**.**001**	8 (6;10) (*n* = 661)	10 (8;11)	**<0**.**001**	0.5179

Data are expressed as median (1st - 3rd quartiles); *n*, numbers; 6MWT, 6-Minute Walking Test; BI, Barthel Index; BId, Barthel Index Dyspnea; CAT, COPD Assessment Test; SPPB, Short Physical Performance Battery.

As shown in Table 3, the proportion of participants reaching the MCID in assessed outcomes was not significantly different between groups. The highest proportion was observed in CAT, whereas the lowest was found in the SPPB.

**Table 3:** Proportion of participants reaching the MCID in outcome measures.

Variable	Non-COPD Bronchiectasis	COPD Bronchiectasis	*P* value
6MWT, %	58.4	54.8	0.438
BId, %	42.1	48.6	0.115
CAT, %	90.9	87.8	0.540
SPPB, %	39.2	46.6	0.308

6MWT, 6-Minute Walking Test; BId, Barthel Index Dyspnea; CAT, COPD Assessment Test; SPPB, Short Physical Performance Battery.

Table 4 shows the correlations between baseline data and the changes in the assessed outcomes. after the program.

**Table 4:** Correlations between baseline values and post program changes in the assessed outcomes.

Variable	Overall	Non-COPD Bronchiectasis	COPD Bronchiectasis
6MWT, m	−0.3233 *P* < 0.001	−0.2412 *P* = 0.0067	−0.3324 *P* < 0.001
BId, score	−0.6916 *P* < 0.001	−0.7428 *P* < 0.001	−0.6782 *P* < 0.001
CAT, score	−0.5306 *P* < 0.001	−0.8172 *P* < 0.001	−0.5157 *P* < 0.001
BI, score	−0.7696 *P* < 0.001	−0.8645 *P* < 0.001	−0.7625 *P* < 0.001
SPPB, score	−0.4934 *P* < 0.001	−0.6871 *P* < 0.001	−0.4798 *P* < 0.001

6MWT, 6-Minute Walking Test; BId, Barthel Index Dyspnea; CAT, COPD Assessment Test; SPPB, Short Physical Performance Battery.

**Figure 2:**
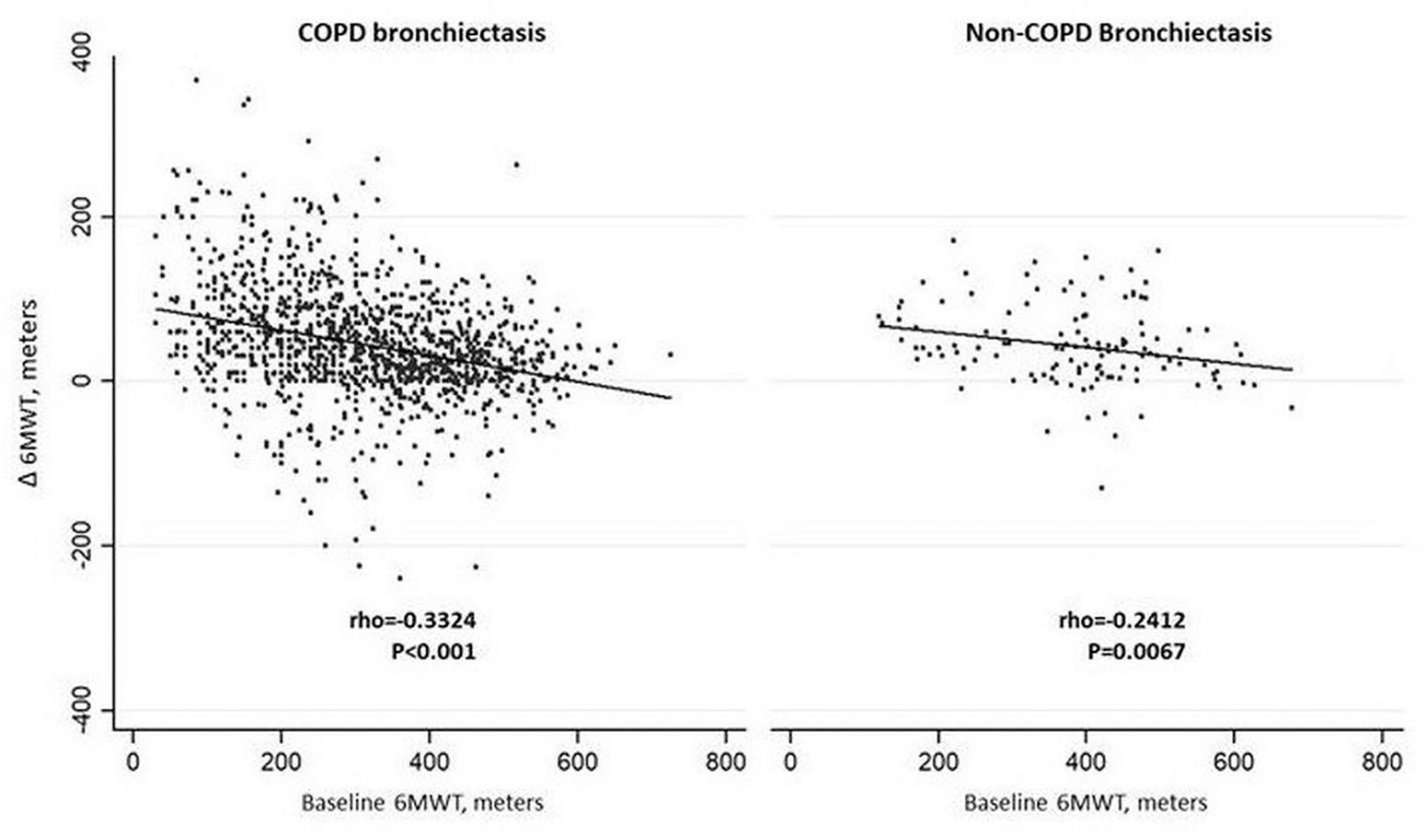
Correlations between baseline 6MWT values (meters) and post-program changes (delta changes in 6MWT). 6MWT, Six-minute walking test.

While 6MWT showed only weak or moderate correlations (see also Figure 2), all other outcomes showed strong correlations as defined in the statistical analysis section.

## Discussion

Our retrospective, multicentric, observational study over a ten-year period shows that in people with non-CF bronchiectasis, pulmonary rehabilitation, including exercise endurance training, is equally effective in improving exercise capacity as assessed by the 6MWT, regardless the association with COPD. All other outcome measures improved significantly in both populations as well. These results suggest the routine clinical provision of pulmonary rehabilitation, including exercise training, to individuals with non-CF bronchiectasis regardless of the concomitant presence of COPD.

Our study adds information on a direct, large, real-life comparison between individuals with and without concomitant COPD. International guidelines recommend pulmonary rehabilitation for people with bronchiectasis, supported by small trials and data extrapolated from COPD. In a real-life, propensity-matched control study, people with bronchiectasis showed similar completion rates and improvements in exercise and health status outcomes as people with COPD (3–5, 7, 28). Our study adds the direct comparison of the two populations of non-CF bronchiectasis.

The prevalence of non-COPD and COPD concomitant non-CF bronchiectasis was 0.54 and 6.31% respectively, in people admitted to our hospitals along the ten year observation. It has been reported that non-CF bronchiectasis affects 0.7% of the general US population, with 20%–51% of cases associated with COPD depending on the setting; among hospitalized individuals with bronchiectasis, COPD is present in up to 39% (3, 8, 29).

Our study confirms that non-CF bronchiectasis without concomitant COPD is more prevalent in women than in men. This female predominance is consistent across large registries in the US, UK, and Europe (3). However, as confirmed by our study, when the disease coexists with COPD, the gender distribution becomes more common in men: multiple studies and meta-analyses demonstrate that the overlap phenotype of COPD and non-CF bronchiectasis is associated with male gender, longer smoking history, and more severe disease (30).

We have used the 6MWT as a measure of exercise capacity and as the primary outcome. While the reliability of this test is well known in people with COPD (10), it has been reported that the 6MWT is a reliable measure of exercise capacity also in non-CF bronchiectasis and responsive to exercise training (31). Our results confirm these observations.

All baseline-assessed outcome measures were significantly worse in people with concomitant COPD, except for the CAT and BI, which did not differ between the two groups. In particular, the 6MWT and the SPPB highlighted a better overall physical condition in people with non-COPD bronchiectasis compared with those with COPD, reflecting superior exercise tolerance, endurance capacity, and muscle strength performance.

There was no difference in baseline BI between groups. This is an expected result as BI is a validated observer-based scale that measures the ability to perform ten basic activities of daily living (ADLs), including feeding, bathing, grooming, dressing, controlling bowel and bladder function, using the toilet, transferring between surfaces (e.g., bed to chair), moving around and climbing stairs. Our participants showed median values around 100 (mean values for the whole population: 88.78 ± 17 vs. 94.35 ± 10.17 at baseline and after the program, respectively), indicating complete independence in the majority of cases. However, a subgroup of participants (19% at baseline and 6.8% after the program) had BI values under 80.

Only dyspnea during ADL, as assessed by BId, improved more in the COPD-related bronchiectasis group. However, the proportion of participants reaching the MCID of BId was not different between the two groups. The reason for this observation remains unclear: a (only) ceiling effect is unlikely, as the correlation between baseline and post-program changes was not stronger than the other outcome measures.

While concomitant COPD adds a health burden in exercise capacity and dyspnea, the health status, as assessed by CAT, seems to be affected mainly by the presence of bronchiectasis. Although data of specific CAT components were unavailable, we can speculate that this might be due to the CAT component cough, phlegm (mucus), and chest tightness, usually relevant in bronchiectasis which might have equalized the effects of worse exercise-induced breathlessness in people with concomitant COPD. Indeed, the highest proportion of participants reaching the MCID in both groups was observed in CAT.

Our study adds also provide useful information on the use of SPPB in bronchiectasis. The SPPB showed the lowest proportion of participants reaching the MCID after the program, with no differences between groups, mainly due to the less severe baseline impairment. The SPPB is a validated, practical tool for assessing physical function in the lower extremities of people with COPD, and its use is growing in the treatment of bronchiectasis. In COPD, the SPPB is responsive to change following pulmonary rehabilitation, predicts mortality, and is associated with risk of hospital admission and LoS for acute exacerbations. While direct studies of the SPPB in bronchiectasis are limited, the pathophysiology —reduced muscle strength and exercise tolerance—mirrors that of COPD, supporting the use of SPPB for functional assessment and rehabilitation monitoring (32, 33).

While 6MWT showed only weak or moderate correlations between baseline values and post-program changes (Table 4), all other outcomes showed relatively stronger correlations. This might indicate that improvements in 6MWT are less dependent on the low baseline values (i.e., a ceiling effect) than the other outcomes. It has been reported that baseline lung function and exacerbation history may predict response to exercise training in these individuals (34).

Our study evaluated the short-term effects of pulmonary rehabilitation. Long-term maintenance of benefits is challenging, as in other conditions, improvements in exercise capacity and health status tend to diminish at 12 months without ongoing intervention (4, 5, 35).

The main limitations of our study, such as missing data, are related to its retrospective design while the long study period might have introduced some heterogeneity factors. Due to the retrospective observational design and the lack of adjustment for potential confounders, causal inferences for example regarding the effectiveness of pulmonary rehabilitation and dyspnea impact during 6MWT might not be assured.

However, our study represents a real-life condition, and its results are supported by the large sample size at a time when even randomized controlled trials are being questioned (36). Diagnoses of bronchiectasis, COPD, CRF, and other comorbidities were based on the ICD-10-CM codes and not on reported physiological measurements such as lung function, arterial blood gases, clinical or physiological assessments, including CT scans. Neither levels of physiological severity (e.g., GOLD classes) were available.

In conclusion, with the above limitations and at least for our cohorts, our study indicates that a multidisciplinary rehabilitation program including endurance training is associated with largely equivalent benefits in exercise capacity and patient-reported outcomes in people with non-CF bronchiectasis with and without COPD. However, improvements in dyspnea during ADL as assessed by BId were greater in those with COPD. The highest proportion of participants reaching the MCID for both groups was observed for the CAT score. Our results suggest the clinical provision of pulmonary rehabilitation to people with bronchiectasis, regardless concomitant COPD.

## Data Availability

Data are avaliable on request to the corresponding author. Requests to access these datasets should be directed to michele.vitacca@icsmaugeri.it.
