# Supplementary material for: Effectiveness of exercise training in people with non-cystic fibrosis bronchiectasis with and without COPD
Source: Front Rehabil Sci. 2026 Mar 6;7:1764160. doi: 10.3389/fresc.2026.1764160 (PMC13002832; doi:10.3389/fresc.2026.1764160)
Supplement: Supplementary file 1 [file Table1.docx]

**Supplementary Material**

**Table 1SM**. Anthropometric, demographic, physiological and clinical characteristics of 2673 patients who suffered from non-CF bronchiectasis admitted during the study period.

|  | **Non-COPD Bronchiectasis** | **COPD**  **Bronchiectasis** | **P value** |
| --- | --- | --- | --- |
| Participants, n (%) | 212 (7.93) | 2461 (92.06) | 0.0127 |
| Male, n (%) | 71 (33.33) | 1356 (55.10) | <0.001 |
| Age, years | 67.82 ± 13.74 | 71.92 ± 10.59 | **<0.0001** |
| BMI, kg/m^2^ | 26.31 ± 5.79 | 25.78 ± 6.63 | 0.6378 |
| LOS, days | 21.99 ± 7.67 | 24.93 ± 10.30 | **<0.0001** |
| From Acute care hospitals, n (%) | 82 (38.67) | 1255 (50.99) | **0.00058** |
| CIRS 2, score | 3.84 ± 2.17 | 3.75 ± 1.97 | 0.8032 |
| Asthma, n (%) | 40 (18.86) | 50 (2.03) | <0.0001 |
| OSAS n (%) | 35 (16.5) | 515 (20.92) | 0.1269 |
| Hypertension, n (%) | 152 (71.69) | 1970 (80.04) | 0.0039 |
| Other CVD, n (%) | 80 (37.73) | 1033 8 (41.97) | 0.2296 |
| Diabetes n (%) | 45 (21.22) | 745 (30.27) | 0.0056 |
| CRF, n (%) | 0 | 861 (34.98) | <0.0001 |
| Pneumonia, n (%) | 2 (0.94) | 40 (1.62) | 0.4436 |
| Night/exercise hypoxaemia, n (%) | 28 (13.20) | 202 (8.24) | 0.0127 |
| CPAP/NIV, n (%) | 29 (13.67) | 499 (20.27) | 0.0206 |
| O_2_ supply, n (%) | 30 (14.15) | 1175 (47.74) | <0.0001 |
| ABG/night pulsossimetry, n (%) | 168 (79.24) | 2187 (88.86) | <0.0001 |
| Lung CT scan, n (%) | 49 (23.11) | 674 (27.38) | 0.1789 |

**Abbreviations**: n=number of available data, ABG = Arterial blood gases; BMI = Body-Mass Index; LOS = Length of Stay; CIRS = Cumulative Illness Rating Scale; CRF = Chronic respiratory failure; CPAP = Continuous Positive Airway Pressure; NIV = Non-Invasive ventilation; O_2_ = Oxygen;. OSAS: Obstructive Sleep Apnoea Syndrome; CVD = Cardiovascular Disease, CT = Computeted tomography. Data shown as mean (SD) or median and 1st - 3rd quartiles
